# Adapting Longstanding Public Health Collaborations between Government of Kenya and CDC Kenya in Response to the COVID-19 Pandemic, 2020–2021

**DOI:** 10.3201/eid2813.211550

**Published:** 2022-12

**Authors:** Amy Herman-Roloff, Rashid Aman, Taraz Samandari, Kadondi Kasera, Gideon O. Emukule, Patrick Amoth, Tai-Ho Chen, Jackton Kisivuli, Herman Weyenga, Elizabeth Hunsperger, Clayton Onyango, Bonventure Juma, Peninah Munyua, Daniel Wako, Victor Akelo, Davies Kimanga, Linus Ndegwa, Ahmed Abade Mohamed, Peter Okello, Samuel Kariuki, Kevin M. De Cock, Marc Bulterys, CDC-Kenya COVID-19 Response Team

**Affiliations:** US Centers for Disease Control and Prevention (CDC), Nairobi and Kisumu, Kenya (A. Herman-Roloff, T. Samandari, G. Emukule, T.-H. Chen, H. Weyenga, E. Hunsperger, C. Onyango, B. Juma, P. Munyua, D. Wako, V. Akelo, D. Kimanga, L. Ndegwa, K.M. De Cock, M. Bulterys);; Ministry of Health, Nairobi (R. Aman, K. Kasera, P. Amoth);; Kenya Prisons Service, Nairobi (J. Kisivuli, P. Okello);; Africa Field Epidemiology Network, Nairobi (A.A. Mohamed);; Kenya Medical Research Institute, Nairobi (S. Kariuki)

**Keywords:** COVID-19, respiratory infections, severe acute respiratory syndrome coronavirus 2, SARS-CoV-2, SARS, coronavirus disease, zoonoses, viruses, coronavirus, Global Health Security Agenda, global health security, PEPFAR, emerging disease outbreak response, bilateral partnership, Kenya, CDC, Centers for Disease Control and Prevention

## Abstract

Kenya’s Ministry of Health (MOH) and the US Centers for Disease Control and Prevention in Kenya (CDC Kenya) have maintained a 40-year partnership during which measures were implemented to prevent, detect, and respond to disease threats. During the COVID-19 pandemic, the MOH and CDC Kenya rapidly responded to mitigate disease impact on Kenya’s 52 million residents. We describe activities undertaken jointly by the MOH and CDC Kenya that lessened the effects of COVID-19 during 5 epidemic waves from March through December 2021. Activities included establishing national and county-level emergency operations centers and implementing workforce development and deployment, infection prevention and control training, laboratory diagnostic advancement, enhanced surveillance, and information management. The COVID-19 pandemic provided fresh impetus for the government of Kenya to establish a national public health institute, launched in January 2022, to consolidate its public health activities and counter COVID-19 and future infectious, vaccine-preventable, and emerging zoonotic diseases.

In December 2019, cases of pneumonia of unknown origin were reported in Wuhan, China (*1*). The disease, COVID-19, was caused by the beta-coronavirus SARS-CoV-2; the World Health Organization (WHO) declared the COVID-19 outbreak a public health emergency of international concern on January 30, 2020, and a pandemic on March 11, 2020 (*2*). By December 23, 2021, a total of 276.4 million persons had received a confirmed SARS-CoV-2–positive test and >5.4 million persons had died from COVID-19 (*3*).

Countries in sub-Saharan Africa, including Kenya, conducted enhanced surveillance activities before their first documented COVID-19 cases (*4*). Early surveillance activities in Kenya used platforms established by Kenya’s Ministry of Health (MOH) and Kenya Medical Research Institute that are supported by external partners, including the WHO, US Centers for Disease Control and Prevention in Kenya (CDC Kenya), US Agency for International Development, US Department of Defense, Wellcome Trust (*5*), and Africa Centres for Disease Control and Prevention. COVID-19 prevention, detection, and response efforts in Kenya began in mid-January 2020 and included laboratory strengthening, deployments to the national public health emergency operations center (PHEOC), training healthcare workers on infection prevention and control (IPC), enhanced surveillance, and screening persons arriving at ports of entry (POE). On March 13, 2020, the president of Kenya announced the country’s first laboratory-confirmed COVID-19 case, which was detected by the National Influenza Center (NIC) Reference Laboratory. By December 26, 2021, Kenya had experienced 5 epidemic waves (July and November 2020 and March, August, and December 2021); a total of 282,554 laboratory-confirmed COVID-19 cases, 5,361 related deaths, and a case fatality rate of 1.9% were reported (Figure 1) (*6*). In addition, 64% of the population in Kibera, a densely populated informal settlement in Nairobi, were exposed to SARS-CoV-2 by June 2021 (*7*).

**Figure 1:**
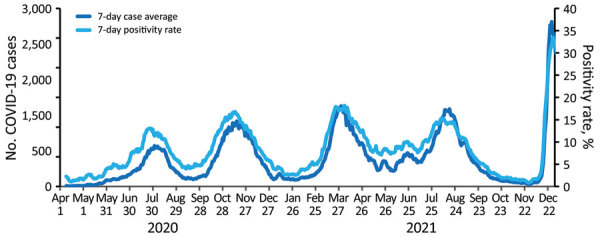
Average number of laboratory-confirmed COVID-19 cases and SARS-CoV-2 positivity rates in Kenya from April 1, 2020, through December 26, 2021, in a review of longstanding public health collaborations between the government of Kenya and CDC Kenya in response to the COVID-19 pandemic. The US Centers for Disease Control and Prevention in Kenya supports the Kenya Ministry of Health with COVID-19 data analysis and visualization. By December 26, 2021, Kenya had experienced 5 epidemic waves during July and November in 2020 and March, August, and December in 2021; a total of 282,554 laboratory-confirmed COVID-19 cases, 5,361 related deaths, and a case fatality rate of 1.9% were reported. The graph shows the 7-day averages for the number of COVID-19 cases and positivity rates.

For >40 years, CDC Kenya has partnered with Kenya’s MOH to support developing workforce capacity, infrastructure, and systems to prevent, detect, and respond to multiple disease threats. We describe longstanding collaborations between CDC Kenya and the government of Kenya that were adapted to respond to the COVID-19 pandemic in 2020 and 2021. In addition, we briefly describe some new initiatives for controlling COVID-19. The experience in Kenya illustrates how existing public health assets can be adapted and mobilized for new epidemics.

## CDC Kenya’s Technical and Financial Assistance to MOH COVID-19 Response

As of December 2021, CDC Kenya had 26 staff from the United States, 124 staff from Kenya, and offices in Nairobi and Kisumu. In January 2020, CDC Kenya formed an internal COVID-19 response team that was aligned to Kenya MOH’s outbreak response pillars: surveillance, laboratory diagnostics, finance and logistics, IPC, clinical care, and emergency management. In March 2020, in coordination with Kenya MOH, CDC Kenya deployed 53 Kenya-based technical experts across the country to support the COVID-19 response (9 staff were US citizens and 44 were Kenya citizens). By December 2021, 32 CDC Kenya staff remained deployed to support Kenya’s COVID-19 response. Upon detection of COVID-19 in Kenya, CDC Kenya allocated funds to implementing partners to support COVID-19 activities through the Global Health Security Agenda, US President’s Emergency Plan for AIDS Relief, and other CDC Kenya programs, such as the Influenza Program. By December 2021, CDC Kenya had awarded nearly $40 million to implement COVID-19 response activities in support of the MOH and 17 select counties in Kenya (Appendix Table).

## Workforce and Emergency Management and Response

The Kenya MOH established PHEOC in 2016 with support from CDC Kenya and WHO. Initial technical and financial support included equipping the physical space, drafting standard operating procedures, and training staff in the incident management system and outbreak response. From 2013 through November 2022, a total of 6 citizens of Kenya attended CDC’s Public Health Emergency Management fellowship training program (*8*), 4 of whom completed the pro¬gram in 2020-2021. The fellowship includes a rotation in the CDC Emergency Operations Center (EOC) in Atlanta, Georgia, USA (*9*). The previous PHEOC incident manager in Kenya completed this fellowship; 2 additional fellows joined Kenya’s COVID-19 response team immediately after completing the fellowship and applied their expertise to finalize PHEOC’s national strategic plan and framework documents. Three more citizens of Kenya participated in the program in 2022.

Since 1980, CDC has trained >18,000 epidemiologists in >80 countries through the CDC Field Epidemiology Training Program (*10*). The Kenya MOH launched the Kenya Field Epidemiology and Laboratory Training Program (FELTP) in 2004, providing training in basic, intermediate, and advanced field epidemiology to 850 graduates over the past 16 years. The MOH relies on FELTP graduates as lead responders during public health emergencies. For example, by the end of 2021, a total of 79% (173/220) of graduates from the 2-year advanced FELTP worked in 40 of Kenya’s 47 counties. During 2020–2021, a total of 22 FELTP residents were deployed to counties to provide mentorship to rapid response teams, and 20 FELTP residents were deployed to POEs to screen 145,275 travelers from >2,000 international flights. The MOH asked CDC Kenya to double the number of residents in the FELTP in 2022 to rapidly expand the workforce. Although FELTP residents were critical assets, early assignments, such as screening travelers, did not optimize their highly technical skill sets. After the government of Kenya increased frontline staff, FELTP residents were able to focus on data analyses and use.

CDC Kenya provided in-person support to Jomo Kenyatta International and Moi International airports and Busia, Malaba, Namanga, Isebania, and Lunga Lunga 1-stop border posts. They trained >1,000 nonhealth workers using a RING (Recognize, Isolate, Notify, and Give support) card as job aid. In June 2020, ≈1,000 truck drivers crossed the Kenya–Uganda border daily, creating a 50-km backup at the border that attracted international media attention (*11*). A coordinated effort by several partners, including CDC Kenya, helped resolve those delays through the deployment of additional human resources and an information system that enabled the MOH to process truckers efficiently, while a CDC Kenya-supported laboratory processed specimens.

A primary responsibility of PHEOC is to integrate data from multiple sources to provide timely information for decision makers. In partnership with the Kenya MOH, CDC Kenya supported the development of PHEOC’s integrated dashboard that displayed outbreak information. In addition, the Kenya MOH guided the adaptation of 3 CDC Kenya-supported information systems: Kenya electronic medical records system (*12*), an online client management system that was expanded from its HIV program support origins to enable COVID-19 case investigation, contact tracing, and management of quarantined contacts; viral load database, an automated HIV laboratory database that was adapted to track COVID-19 real-time reverse transcription PCR (RT-PCR) testing, cycle threshold values, and indirectly track commodities; and Jitenge, a mobile application that enabled POE travelers and quarantined clients to self-report daily on their health status in a national database (*13**)*.

**Figure 2:**
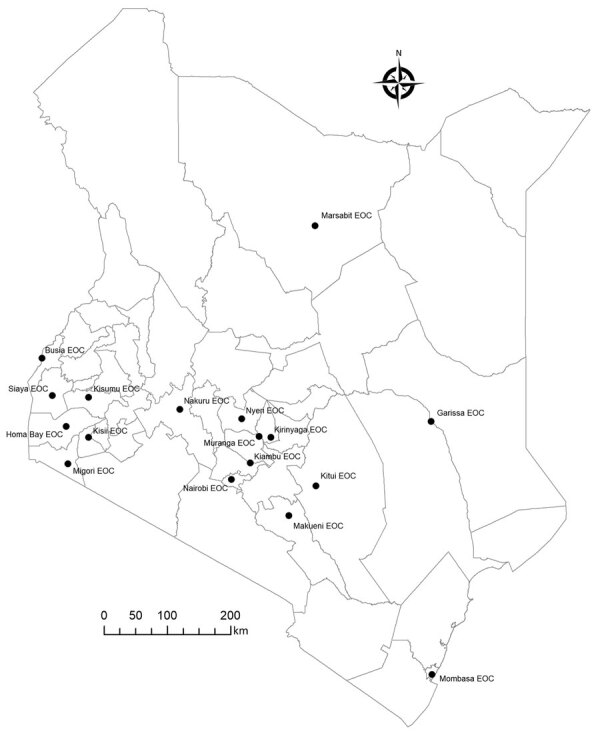
Locations of county EOCs supported by CDC Kenya in a review of longstanding public health collaborations between the government of Kenya and CDC Kenya in response to the COVID-19 pandemic. By early 2021, the Kenya Ministry of Health, US Centers for Disease Control and Prevention in Kenya, and World Health Organization began decentralizing Kenya’s emergency management. As of December 2021, a total of 17 county-led EOCs had been established in Kenya. Those 17 new EOCs established incident management systems and produced routine situation reports that guided the county-level response to the COVID-19 epidemic in Kenya. EOC, Emergency Operations Center.

By early 2021, the Kenya MOH, supported by CDC Kenya and WHO, began decentralizing emergency management and established county-led EOCs. With initial seed funding of $750,000 from CDC Kenya, those 17 new EOCs established an incident management system and produced routine situation reports that guided the county-level response (Figure 2). County EOCs managed local outbreaks, including implementing isolation and quarantine measures during the SARS-CoV-2 outbreak. The EOCs coordinated their COVID-19 vaccination campaigns, tracing thousands of persons who did not report for their second vaccine, and integrated programs to improve uptake among clinically vulnerable populations, such as persons living with HIV.

## Diagnostic Laboratory Support

Building capacity for laboratory diagnostics has been a pillar of the Kenya MOH and CDC Kenya partnership (*14*). WHO emphasized that timely SARS-CoV-2 testing was the foundation of each country’s response (*15*). In the early stages of the COVID-19 epidemic in Kenya, the MOH focused on testing persons returning from international travel. However, once local transmission was well-established in mid-2020, after which >50% of patients had no international travel history, the MOH adopted a strategy to use human resources for tracing contacts and testing persons in high-risk settings, such as healthcare workers.

RT-PCR remains the definitive assay to detect SARS-CoV-2 RNA in Kenya. National laboratory diagnostics for the COVID-19 response were coordinated by the NIC, which was established in 2017 partly through support from CDC Kenya, who aided in the procurement and installation of equipment, preparation for WHO accreditation (granted in 2019), and building staff capacity for respiratory pathogen testing using RT-PCR. Coordination of partners by the Kenya MOH enabled the NIC to achieve testing capacity for SARS-COV-2 in early February 2020. The Africa Centres for Disease Control and Prevention procured RT-PCR primers and reagents, CDC Kenya collaborated with NIC staff to validate the assay, and WHO confirmed Kenya’s testing capacity within 24 hours of receiving the reagents. Throughout the COVID-19 pandemic, the NIC coordinated the diagnostics of 50 laboratories that test for SARS-CoV-2.

CDC Kenya’s laboratory support focused on commodity and personal protective equipment (PPE) procurement, specimen transport, test verification, salary support for additional laboratory personnel, genomic sequencing, and repurposing high-throughput RT-PCR–based HIV testing platforms for SARS-CoV-2. CDC Kenya supported 45% of SARS-CoV-2 testing in Kenya in 2020, decreasing to 34% in 2021 after additional MOH laboratories were established. Also in 2021, Kenya MOH and CDC Kenya collaborated on a field evaluation of an antigen rapid diagnostic test (RDT) kit, and 2 CDC-supported laboratories were among the 5 laboratories performing genomic sequencing. The CDC-supported laboratory in Kisumu was the first to detect the SARS-CoV-2 Delta variant in Kenya at the beginning of the fourth wave; sequencing results were used for the first time to inform mitigation measures coordinated by the county EOC, including quarantine and isolation measures and intensified case and contact tracing.

## Epidemic Intelligence

Kenya MOH and CDC Kenya have collaborated on surveillance initiatives for >2 decades. In January 2020, leveraging surveillance platforms for early detection and mitigation of SARS-CoV-2 was identified as a priority activity by the MOH. CDC’s surveillance platforms are protocol-driven and including COVID-19 testing required an amendment. To improve the responsiveness of these platforms in the future, CDC Kenya added language to health security protocols that increase flexibility when the PHEOC is activated.

CDC Kenya’s longstanding support of several surveillance systems created geographically diverse opportunities for COVID-19 monitoring. Kenya first implemented severe acute respiratory illness surveillance in 2006 (*16*); the platform currently operates in 8 sites that receive CDC Kenya support to conduct active hospital-based surveillance. By December 2021, a total of 5,162 patients had samples tested for SARS-CoV-2 of which 509 were positive (9.9% positivity rate). Also, since 2006, Kenya Medical Research Institute and CDC Kenya have conducted population-based infectious disease surveillance (PBIDS) in Kibera in Nairobi County, one of the largest informal urban settlements in Africa, and rural Asembo in Siaya County (*17*,*18*). The PBIDS platform includes health facility and household components for a population of >25,000 persons per site. The first COVID-19 case in Kibera was detected on May 8, 2020, by using the PBIDS platform. By December 2021, a total of 1,572 cases (14.0% positivity rate) in Kibera and 628 cases (6.1% positivity rate) in Asembo were reported. Furthermore, in Kibera, CDC Kenya supported 3 rounds of a SARS-CoV-2 seroprevalence survey. The overall weighted individual seroprevalence increased from 43.3% (95% CI 37.4%–49.5%) in December 2020 to 63.9% (95% CI 59.5%–68.0%) in June 2021 (*7*). In 2019, CDC Kenya supported the introduction of a pilot event-based surveillance platform in 4 counties. Similar to the Ebola virus disease response in Guinea (*19*), the core feature of event-based surveillance was a toll-free telephone line to report unusual events for investigation. Activity on the toll-free line reached a peak of 100,000 calls/day after the onset of the COVID-19 epidemic in Kenya.

**Figure 3:**
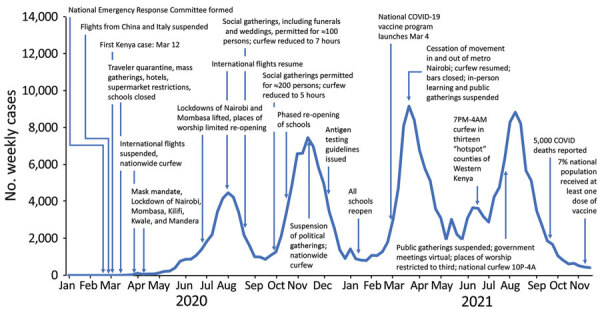
Government applied nonpharmaceutical interventions during the COVID-19 pandemic in Kenya from February 2020 through November 2021 in a review of longstanding public health collaborations between Government of Kenya and CDC Kenya in response to the COVID-19 pandemic. Examples of nonpharmaceutical interventions utilized in Kenya include traveler quarantine, restrictions on mass gathering, school closures, mask mandates, curfews, and phased lifting of restrictions in response to case levels. The graph indicates the number of weekly cases and the period in which the specific nonpharmaceutical and pharmaceutical interventions were implemented.

CDC Kenya supports Kenya MOH with data analysis and visualization (Figure 1), including monitoring populations of interest such as truck drivers and healthcare workers. CDC Kenya participates in the MOH-directed COVID-19 national task force and contributes substantially to the modeling committee that generates analyses briefs for policy makers, including recommendations for implementing nonpharmaceutical interventions. For example, during the first COVID-19 wave, all patients were directed to isolate in facilities, and travel between all 47 counties was suspended, regardless of county epidemiology. The MOH rapidly revised guidance to enable patients with mild COVID-19 to recover at home. During the third wave, when most transmissions occurred in 5 counties, the MOH prohibited travel in and out of those counties, while leaving travel open for residents in the remaining 42 counties. Additional examples of nonpharmaceutical interventions used in Kenya included traveler quarantine, mass gathering restrictions, school closures, mask mandates, curfews, and phased lifting of restrictions in response to case levels (Figure 3). In March 2021, one year after the first COVID-19 case was detected in Kenya and in the absence of available data visualization, CDC Kenya partnered with WHO and Kenya MOH to launch a public dashboard that integrated and displayed COVID-19 epidemic information to support data-informed decision making. This dashboard filled a gap and was used to guide the acute response phase; the country now relies on daily situation reports and the WHO COVID-19 dashboard (*3*).

## County Engagement and Response in Kisumu County

In 2019, CDC Kenya assisted the Kisumu County Department of Health in conducting an outbreak readiness assessment in the event that the Ebola virus outbreak in the Democratic Republic of the Congo spread to Kenya. This assessment exercise established a foundational partnership before the first COVID-19 case was detected in Kisumu County in June 2020. Compared with Nairobi County, Kisumu County had 3 extra months to prepare for COVID-19 cases and conducted mass procurement of PPE and test kits. CDC Kenya and Kisumu County Department of Health implemented the WHO’s First Few X Cases protocol used to track community transmission among the first 150 cases and their close contacts (*20*). In addition, CDC Kenya supported the installation of the county EOC; trained staff on the incident management system; equipped the EOC physical space; developed guidance on PPE use, waste management, patient flow modifications, and facility and isolation preparedness assessments; and participated in community education opportunities. In May 2021, the Kisumu EOC coordinated the initial response to the first cases of the SARS-CoV-2 Delta variant in Kenya with support from FELTP residents.

## Health System

The Kenya MOH partnered with CDC Kenya to establish the patient and health worker safety unit >10 years ago. CDC Kenya and WHO supported the MOH’s IPC guideline development, training, and policy development and dissemination. One of CDC Kenya’s major activities during 2020–2021 was providing technical and financial support to train >8,500 MOH, rapid response team, POE, county health, and healthcare workers on screening procedures, IPC, case and contact tracing, risk communication, sample collection, and biosafety and biosecurity. Biosafety and biosecurity scored particularly low in the 2017 WHO Joint External Evaluation (*21*). Training was adapted to a virtual environment in February 2020, and virtual components became available on the Project ECHO platform (*22*) to all 47 counties by September 2021.

For ≈2 decades, CDC Kenya provided technical assistance to the Kenya Prisons Service. Because of prison overcrowding, Kenya MOH prioritized testing inmates for SARS-CoV-2 to improve early identification and case management. By December 2021, a total of 10,925 cases of COVID-19 were identified among staff and 85,229 inmates. Test positivity rates of up to 90% were observed during outbreaks among new inmates in some prisons. In 2020, CDC Kenya worked with Kenya Prisons Service to develop COVID-19 control plans, integrate COVID-19 and tuberculosis screening for new inmates, train employees on IPC, and analyze data. In 2021, CDC Kenya and Kenya Prisons Service launched an EOC to help control COVID-19 and other outbreaks in prison settings, and, by December 2021, 94.5% of inmates had received at least one COVID-19 vaccine dose (Kenya Prisons Service, pers. comm., email, 2021 Dec 26).

The Kenya MOH focused on COVID-19 vaccine distribution after administering the first dose on March 5, 2021. As of December 24, 2021, a total of 5.6 million persons had received their first dose, and 3.9 million persons were fully vaccinated (14.4% of the goal) (*6*). Of the 23.3 million vaccine doses donated to Kenya by December 2021, the US government donated 7.4 million doses (31.8%) through the COVAX (COVID-19 vaccines global access) initiative and was the largest bilateral donor of COVID-19 vaccines. In late 2020, the government of Kenya established a multisectoral COVID-19 vaccination deployment task force to develop a national vaccination deployment plan that prioritized certain groups to receive the vaccine, including healthcare workers, teachers, security personnel, and persons >58 years of age. By September 2021, CDC Kenya and partners trained >2,500 healthcare workers on vaccine administration in 8 priority counties, which comprised 26% of the national vaccination objective. CDC Kenya also supported Kenya’s Pharmacy and Poisons Board to strengthen passive and active surveillance of adverse events following immunization.

## Discussion

By December 2021, Kenya had experienced 5 COVID-19 epidemic waves. Kenya MOH leveraged its partnership with CDC Kenya, and the multidisciplinary CDC Kenya response team was deployed to support the COVID-19 response. The country’s COVID-19 Intra-Action Review noted that Kenya MOH’s ability to adapt and utilize longstanding health security activities for the COVID-19 response was a strength (S.H. Matendechero, Kenya MOH, pers. comm., email, 2021 Dec 7). Examples of those activities were incorporating SARS-CoV-2 testing into existing surveillance platforms, strengthening the national PHEOC while installing 17 county-level EOCs, deploying 42 FELTP residents across the country to contribute to response activities, using CDC Kenya–supported laboratories to perform 45% of nationwide COVID-19 testing in 2020 and 34% of testing in 2021, and using the PBIDS platform to detect the first case in Kibera.

Because of the demonstrated importance of workforce development and the PHEOC during the COVID-19 pandemic, in August 2020, the president of Kenya announced that the government would establish a national public health institute (NPHI). The institute would integrate essential public health pillars that were already performing well according to the WHO Joint External Evaluation score (Appendix Table) (*21*); surveillance and laboratory platforms would be connected further to enable the national PHEOC and county EOCs to monitor and respond to diseases of public health interest in a timely and appropriate manner. The NPHI was legally launched in January 2022, and its design and resources are being established by the government of Kenya. 

Although laboratory capacity and epidemiologic surveillance form the foundation of a public health response (*23*), Kenya was chronically on the verge of running out of laboratory commodities during most of 2020. Relying heavily on donor procurement during 2020, the MOH used 15 different test kits, which met the testing demand but also complicated commodity management. Until testing eased between waves, the Kenya MOH promoted commodity sharing between laboratories according to test platforms and volume, which was monitored by the viral load database. Considering these realities, the MOH and CDC Kenya concluded a field evaluation of an antigen RDT kit in July 2021 that demonstrated adequate sensitivity only among symptomatic patients who had high viral loads (*24*). Those unexpected results were used by Kenya MOH to clarify that antigen RDTs should be used in high-transmission settings or in locations where RT-PCR testing was not easily accessible, and negative RDT results should be confirmed by RT-PCR.

Although CDC Kenya contributed to Kenya’s health security, the MOH’s routine coordination of donors and partners through the national task force and the Development Partners for Health in Kenya was identified as a strength during the COVID-19 Intra-Action Review (S.H. Matendechero, Kenya MOH, pers. comm., email, 2021 Dec 7). This coordination continues to be essential to ensure that dedicated COVID-19 resources are used appropriately and critical areas are optimally managed. As vaccine distribution has increasingly become the focus, broad national task force coordination has decreased in frequency, which has resulted in reduced management of communication, laboratory, and surveillance functions. The COVID-19 Vaccination Intra-Action Review identified the need to strengthen human resources, cold chain capacity, and surveillance data management to optimize vaccine distribution (S.H. Matendechero, Kenya MOH, pers. comm., email, 2021 Dec 7).

The COVID-19 response has provided the Kenya MOH–CDC Kenya partnership the opportunity to evolve. The 2017 WHO Joint External Evaluation (*21*) noted that CDC Kenya predominantly financed the FELTP. CDC Kenya and Kenya MOH are committed to shared support for this program, and the MOH increased its funding for the FELTP, while also seeking additional funding from the Global Fund. Joint financial ownership has led to improved collaboration, including reviewing the FELTP curriculum for health informatics in response to the COVID-19 Intra-Action Review findings that there were inadequate electronic data management systems in Kenya (S.H. Matendechero, Kenya MOH, pers. comm., email, 2021 Dec 7).

The main limitation of our review is that some aspects of program monitoring and vaccine defaulter tracing were constrained because health information systems were not interoperable. For example, Chanjo KE, Kenya’s COVID-19 vaccine registration system, was not interoperable with other systems used by the MOH and PHEOC. Emerging from the COVID-19 pandemic, the Kenya MOH is pursuing an ambitious vision for a digital health platform, a clinical system built on interoperable modules linked by a unique patient identifier. This system would link laboratory and clinical data enabling improved program and patient monitoring.

Containing the pandemic will require continued and increased coordination by national and county leadership to maintain increased vaccination coverage, improve access to testing, ensure quality healthcare availability, and use nonpharmaceutical interventions wisely (*25*,*26*). The activities implemented by Kenya MOH and CDC Kenya over the past 4 decades were adapted and used to strengthen the COVID-19 response, which focused specifically on 5 core capabilities of CDC: data and analytics, laboratory capacity, public health expertise, outbreak response, and global capacity building (*27*). As Kenya establishes its NPHI, along with support from WHO and the Africa Centres for Disease Control and Prevention, CDC Kenya’s partnership with the MOH will continue to reinforce ongoing efforts to prepare for and respond to health threats in the country and region.

Appendix Additional information for adapting longstanding public health collaborations between Government of Kenya and CDC Kenya in response to the COVID-19 pandemic, 2020–2021.
