# Supplementary material for: Adapting Longstanding Public Health Collaborations between Government of Kenya and CDC Kenya in Response to the COVID-19 Pandemic, 2020–2021
Source: Emerg Infect Dis. 2022 Dec;28(Suppl 1):S159–67. doi: 10.3201/eid2813.211550 (PMC9745212; doi:10.3201/eid2813.211550)
Supplement: Appendix — Additional information for adapting longstanding public health collaborations between Government of Kenya and CDC Kenya in response to the COVID-19 pandemic, 2020–2021. [file 21-1550-Techapp-s1.pdf]

Article DOI: https://doi.org/10.3201/eid2813.211550

Adapting Longstanding Public Health Collaborations between Government of Kenya and CDC Kenya in Response to the COVID-19 Pandemic, 2020–2021

Appendix

Appendix Table. Summary of CDC Kenya's support of the Government of Kenya's COVID-19 response, January 2020–December 2021, in a review of longstanding public health collaborations between Government of Kenya and CDC Kenya in response to the COVID-19 pandemic*

Domain	Score (Indicator)†	CDC Kenya supported activities
Workforce development	4 (D.4.2)	Deployed FELTP residents across Kenya and at ports of entry
Emergency management and response, national PHEOC	2 (R.2.1)	Provided technical support to the national PHEOC
	NA‡	Trained and mentored PHEOC staff, including four senior staff enrolled in a CDC headquarter-based fellowship program
	2 (R.2.2)	Drafted and launched national PHEOC strategic plan and framework documents in 2021
	4 (D.2.3)	Supported data management, analysis, and visualization through the Emergency Alert and Response System
	2 (R.2.4)	Supported COVID-19 contact tracing
Emergency management and response, ports of entry	2 (PoE.1)	Assessed the preparedness of multiple air, land, and sea ports of entry, including the flagship Jomo Kenyatta International Airport
	4 (D.4.2)	Deployed 20 FELTP residents to key ports of entry to support capacity building and surveillance activities
	2 (PoE.1)	Trained 1,000 nonhealthcare workers on COVID-19 infection prevention and control at ports of entry
	2 (PoE.2)	Supported truck driver registration and quick response code production for laboratory certification of negative SARS-CoV-2 test, and implemented the Jitenge module for truck drivers to report symptoms and receive test results after entry into Kenya
Laboratory	4 (D.1.1)	Provided technical support to the National Influenza Center to coordinate SARS-CoV-2 testing across 50 laboratories and directly conducted $\geq$34% of national testing
	NA	Procured commodities, including PPE, test kits, and reagents
	2 (D.1.2)	Mapped specimen transport networks and supported transport costs
	2 (D.1.3)	Performed new test evaluations and verification
	3 (D.1.4)	Conducted external quality assurance activities
	3 (P.6.2)	Provided biosafety and biosecurity training
		Conducted genomic sequencing in 2 laboratories
	3 (D.4.1)	Provided direct salary support for additional laboratory technicians
Epidemic intelligence, surveillance	4 (D.2.4)	Supported the SARI surveillance system which is operational at 8 sites. SARI surveillance officers trained and supported rapid response teams; 3 counties trained >900 healthcare workers
	4 (D.2.4)	Supported the PBIDS platform, which is operational in Nairobi and Siaya. PBIDS conducted testing in 2 health facilities, trained front line staff, disseminated information about COVID-19, conducted SARS-CoV-2 seroprevalence surveys and knowledge, attitude and practices surveys, and developed standard operating procedures for PPE use
	4 (D.2.1)	Supported event-based surveillance in select counties and the toll-free mobile phone line to identify unusual cases or outbreaks in communities or hospitals; calls reached 100,000/day during the pandemic
	4 (D.2.1)	Implemented rapid mortality surveillance in 6 counties (3 counties were financially supported by CDC Kenya) to improve capture and processing of mortality data and monitor mortality trends in near real-time
Epidemic intelligence, health information systems	2 (D.2.2)	Launched the Emergency Alert and Response System to integrate and display all outbreak information in the PHEOC in near real-time

Domain	Score (Indicator)†	CDC Kenya supported activities
	NA	Launched Jitenge, an automated contact tracing and traveler monitoring system, for travelers and truck drivers
County engagement	NA	Deployed CDC Kenya technical staff to provide direct support to county EOCs in 17 counties serving 48% of the population in Kenya
	NA	Identified and equipped space for 17 new county EOCs
	4 (D.4.2)	Deployed 23 FELTP residents to support county EOCs
Health system, COVID-19 vaccine distribution	NA	Trained 2,500 healthcare workers on vaccine administration and reporting
	NA	Implemented a multi-site knowledge, attitudes, and practices survey on vaccine acceptability and uptake and supported using the results to increase vaccine uptake and acceptability
	1 (R.4.1)	Supported rapid result initiative events in select counties to rapidly increase vaccine coverage
	NA	Supported the COVID-19 Vaccination Intra-Action Review to identify and document best practices and challenges encountered during the first phase of COVID-19 vaccination rollout in Kenya
	NA	Supported the improvement of passive surveillance and development of active surveillance capacity for adverse events following immunization
Health system, infection prevention and control	3 (P.3.3)	Supported and co-authored guidelines for infection prevention and control and the rational use of PPE
	3 (P.3.3)	Conducted facility preparedness assessments in 777 facilities with subsequent training and mentorship provided to improve infection prevention and control
	NA	Adapted training activities to a virtual environment through the installation of Project ECHO in 16 counties; the University of Nairobi served as the superhub; by the end of 2021, Project ECHO was being used to provide training in all 47 counties
	NA	Supported the launch of the healthcare worker case management hotline
	NA	Supported the design and installation of outdoor triage spaces
Health system, clinical management	NA	Supported and co-authored national guidelines for clinical care, care in quarantine and isolation facilities, and home-based care
	NA	Supported and co-developed job aids for clinical staff, supply chain management, considerations for special populations (e.g., pregnant women) and prevalent health risks (e.g., malaria)

*The World Health Organization Joint External Evaluation (JEE) tool is a component of the International Health Regulations (IHR) (2005) Monitoring and Evaluation Framework (https://www.who.int/publications/i/item/9789240051980). The JEE is a voluntary, multisectoral, comprehensive process to evaluate country capacity to prevent, detect, and rapidly respond to public health risks, is in line with required IHR (2005) core capacities, and was used to score capacity in Kenya. The last JEE in Kenya was completed in 2017 before the COVID-19 pandemic. CDC, Centers for Disease Control and Prevention; ECHO, extension for community healthcare outcomes; EOC, emergency operations center; FELTP, Field Epidemiology and Laboratory Training Program; NA, not applicable; PBIDS, population-based infectious disease surveillance; PHEOC, public health emergency operation center; PPE, personal protective equipment; SARI, severe acute respiratory illness.
†Scores for each indicator are: 1, no capacity; 2, limited capacity; 3, developed capacity; 4, demonstrated capacity; and 5, sustainable capacity. Indicators are in accordance with those used in the second edition JEE tool.
‡NA indicates no relevant JEE score was available for this activity.
